# Predictors of oesophageal candidiasis in persons with or without HIV infection

**DOI:** 10.4314/ahs.v18i2.32

**Published:** 2018-06

**Authors:** Felix Bongomin, Samuel A Fayemiwo

**Affiliations:** 1 Division of Infection, Immunity and Respiratory Medicine, The University of Manchester, Manchester, M13 9PL, UK; 2 Department of Medical Microbiology and Immunology, Faculty of Medicine, Gulu University, P.O. Box 166, Gulu, Uganda; 3 University College Hospital, Department of Medical Microbiology and Parasitology, College of Medicine, Ibadan, Nigeria

Dear Sir, We read with great interest the article by Mushia nd colleagues recently published in African Health Sciences^1^. The authors present data on predictors of endoscopically diagnosed oesophageal candidiasis (OC). Much as this is an interesting and one of the largest studies of its kind in the region, it is confusing in one respect. A major distinction between predictors of OC in HIV-infected patients and risk factors for development of OC in HIV-uninfected individuals should have been drawn in the abstract. Additionally, HIV which is a major risk factor for oesophageal candidiasis is missing in the conclusions, and its odds ratio is not only inconsistent with that provided in Table 2 of the results section, but also out of the 95% confidence interval.

OC (or Candida oesophagitis) is the most common infectious disease of the oesophagus; it is an opportunistic infection that complicates disorders associated with granulocyte and/or lymphocyte numbers and dysfunction^2^,^3^. OC is an AIDS-defining illness occurring in patients with advanced HIV disease and is estimated to occur in at least 5% of patients who are on antiretroviral therapy (ART) and in 20% who are ART-naïve^4^,^5^. Predictors of OC in HIV-infected patients include low CD4 counts (typically <50 cells/µL), high HIV RNA viral load , oropharyngeal candidiasis and ART naivety^6^–^8^.

There is no published data on predictors of OC in HIV-uninfected patients. However, risk factors for the development of OC in HIV negative from several published studies are as summarised in Table 1.

**Table 1:** risk factors for the development of oesophageal candidiasis 1 in HIV-infected and HIV-uninfected patients

Major risk factors	Other risk factors
HIV/AIDS Low CD4 counts High HIV-RNA viral load ART naïve patients Oropharyngeal candidiasis Cancer Irradiation (radiotherapy) Chemotherapy Diabetes Hyperglycaemia Immunodeficiency Long-term broad-spectrum oral antibiotic use Proton pump inhibitors Transplant recipients on chemotherapy Presence of oral candidiasis (in children)	Smoking Alcoholism Reflux esophagitis Inhaled corticosteroid use Xerostomia (inadequate saliva) Chronic diseases (liver cirrhosis, chronic heart failure etc.) High sugar diet Increasing age Previous gastric surgeries Oral steroid therapy Defective neutrophil motility

Despite the retrospective nature of the study, the authors have shown some important association between oesophageal candidiasis and risk factors such as diabetes and antibiotic use, though decreasing age as a risk factor as mentioned by the authors in the abstract contradicts their own data provided in Table 2 and established evidence^9^. Summary Table 2 requires rigorous statistical adjustments and improvement on data presentation.

Hence, we regret that the study does not live up to its title and has failed to inform the readers on the predictors of OC both in HIV-infected and HIV-uninfected individuals; it is “a prevalence and factors associated with oesophageal candidiasis” study, as suggested in the aim rather than “predictors of oesophageal candidiasis” as currently stated in the title.
